# Avian Mycobacteriosis and Molecular Identification of *Mycobacterium avium* Subsp. *avium* in Racing Pigeons (*Columba livia domestica*) in Greece

**DOI:** 10.3390/ani11020291

**Published:** 2021-01-24

**Authors:** Vasilios Tsiouris, Konstantinos Kiskinis, Tilemachos Mantzios, Chrysostomos I. Dovas, Natalia Mavromati, Georgios Filiousis, Georgia Brellou, Ioannis Vlemmas, Ioanna Georgopoulou

**Affiliations:** 1 Unit of Avian Medicine, School of Veterinary Medicine, Faculty of Health Sciences, Aristotle University of Thessaloniki, 54627 Thessaloniki, Greece; kiskinik@vet.auth.gr (K.K.); mantzios@vet.auth.gr (T.M.); nataliem@vet.auth.gr (N.M.); ioannag@vet.auth.gr (I.G.); 2 Diagnostic Laboratory, School of Veterinary Medicine, Faculty of Health Sciences, Aristotle University of Thessaloniki, 54627 Thessaloniki, Greece; dovas@vet.auth.gr; 3 Laboratory of Bacteriology and Infectious Diseases, School of Veterinary Medicine, Faculty of Health Sciences, Aristotle University of Thessaloniki, 54627 Thessaloniki, Greece; biltsiou@gmail.com; 4 Laboratory of Pathology, School of Veterinary Medicine, Faculty of Health Sciences, Aristotle University of Thessaloniki, 54627 Thessaloniki, Greece; mprellou@vet.auth.gr (G.B.); ivlemmas@vet.auth.gr (I.V.)

**Keywords:** avian mycobacteriosis, racing pigeon, granulomas, *Mycobacterium avium*

## Abstract

**Simple Summary:**

Avian mycobacteriosis a contagious, chronic and potential zoonotic List B disease of the World Organization for Animal Health, is described in two lofts of pigeons in this review. Molecular analysis identified the causative agent as Mycobacterium avium subsp. avium. This is the first case report of avian mycobacteriosis in Greece, which describes the presence of granulomatous conjunctivitis and the molecular identification of *M. avium* subsp. *avium* as the causative agent in racing pigeons. The identification of the strain will enrich the epidemiological data and will contribute to the control of avian mycobacteriosis in pigeons.

**Abstract:**

In this report, cases of avian mycobacteriosis in two lofts of racing pigeons are described. Three racing pigeons of 2-year old from the first loft (A) and four racing pigeons of 4–5 years old from the second loft (B) were submitted to the Unit of Avian Medicine for clinical examination and necropsy. In the case history chronic and debilitating disease was reported. The clinical signs included emaciation, depression, lameness, periorbital swelling and diarrhea, although the appetite was normal. Post mortem lesions involved an enlarged spleen with multiple different sized yellow nodules. Similar lesions were also observed in the liver, conjunctiva of the inferior eyelids and in the femoral bone marrow. The suspicion of avian mycobacteriosis was based on history, clinical signs and typical lesions. In order to confirm the diagnosis, histopathology was performed on tissue sections and revealed the presence of multiple granulomas with central necrosis. In addition, Ziehl-Neelsen positive bacilli were observed in histological sections and smears from the granulomas of the affected tissues. Molecular analysis identified the causative agent as *Mycobacterium avium* subsp. *avium*. This is the first case report of avian mycobacteriosis in Greece, which describes the presence of granulomatous conjunctivitis and the molecular identification of *M. avium* subsp. *avium* as the causative agent in racing pigeons.

## 1. Introduction

Avian mycobacteriosis is an important chronic, contagious and potential zoonotic disease mainly affecting poultry or captive birds [1]. Τhe causal agent belongs to the *Mycobacterium avium species* which contains four subspecies *Mycobacterium avium* subsp. *avium* (MAA), *Mycobacterium avium* subsp. *hominissuis* (MAH), *Mycobacterium avium* subsp. *paratuberculosis* (MAP) and *Mycobacterium avium* subsp. *silvaticum* (MAS) [2].

A genetically distinct species *M. genavense* (MG) can also cause disease with similar clinical and histopathological findings to that of *Mycobacterium avium* members [2]. However, avian Mycobacteriosis in Columbiformes is most frequently caused by infection with MAA serotypes 1, 2, 3 which are fully virulent and less by MG species [2,3]. MAP and MAS subspecies can cause avian mycobacteriosis in wood pigeon. *M. tuberculosis* and *M. bovis,* which are responsible for true tuberculosis in animals, cause less frequently similar lesions to birds [4,5,6].

*Mycobacterium avium* can potentially cause avian mycobacteriosis in all bird species. It is most prevalent in domestic chickens, sparrows, pheasants, partridges and wild birds in captivity, whereas guinea fowls, turkeys and domestic pigeons seem to be less susceptible in infection [1]. Infections from *Mycobacterium avium* members occur in mammals including swine, sheep, mink, cattle, horse, monkey, cats, dogs and rarely humans [6]. Members of *Mycobacterium avium* are classified in Risk Group 2 for human infection and should be handled with appropriate measures [2].

The most important source of infection is the faeces of the infected birds, which contain large numbers of *Mycobacterium avium* and contaminate the soil, the water and the litter. *Mycobacterium avium* may persist in the soil for up to 4 years [1]. The spread of the bacteria by shoes, equipment and materials as well as by cannibalism might play a role in the transmission of the disease [6].

The clinical signs are not specific and vary depending on the infected organs involved. They may be prolonged over a period of weeks or months before death. The clinical signs include progressive but slow loss of weight and condition, emaciation, lameness and diarrhea. Although the appetite is usually maintained, there is eventually gross emaciation with marked atrophy of the pectoral muscles. Affected birds die in a few months or some birds die suddenly in good body condition due to an internal hemorrhage from the rupture of the liver or the spleen [6].

The lesions of mycobacteriosis in birds are irregular grey to yellow nodules mainly found in the liver, spleen and intestine. Ovary, testes, heart, skin, conjunctiva, lungs and bone marrow may be also affected [7,8,9,10,11,12].

The infected birds can be recognized by immunological tests, such as the tuberculin test, the agglutination test and by ELISA. In pigeons ELISA and rapid agglutination test are not very sensitive, however highly specific [13]. The studies conducted so far have not confirmed the diagnostic significance of the tuberculin test in pigeons [14]. A culture to identify the bacillus would be helpful but not essential for diagnosis [15]. The observation of the acid-fast bacilli in the Ziehl-Neelsen stained smears or histologic sections of tissue samples is the gold standard method for the diagnosis of avian mycobacteriosis. Molecular methods are important for the identification and taxonomy of the causative agent [1]. Insertion elements in the mycobacterial genome permit the determination of *M. avium* subspecies [16,17]. MAA is characterized by the presence of 2–17 copies of IS901 insertion sequence [18,19] and a single copy of IS1245 [20]. MAA and MAS are so close genetically related that the development of a reliable molecular identification method to distinguish between the two lineages was only recently achieved [21].

In practice, the diagnosis of avian mycobacteriosis in pigeons is based only on the gross lesions and Ziehl-Neelsen staining, with no isolation and identification of the causative agent. However, the identification of the species and subspecies is important for the control of avian mycobacteriosis in pigeons as well as for the risk assessment of public health. Therefore, the objective of the present study was to identify the causative agent of the granulomatous lesions observed in conjunctiva, spleen, liver and bone marrow in 2 flocks of racing pigeons.

## 2. History

**Figure 1:**
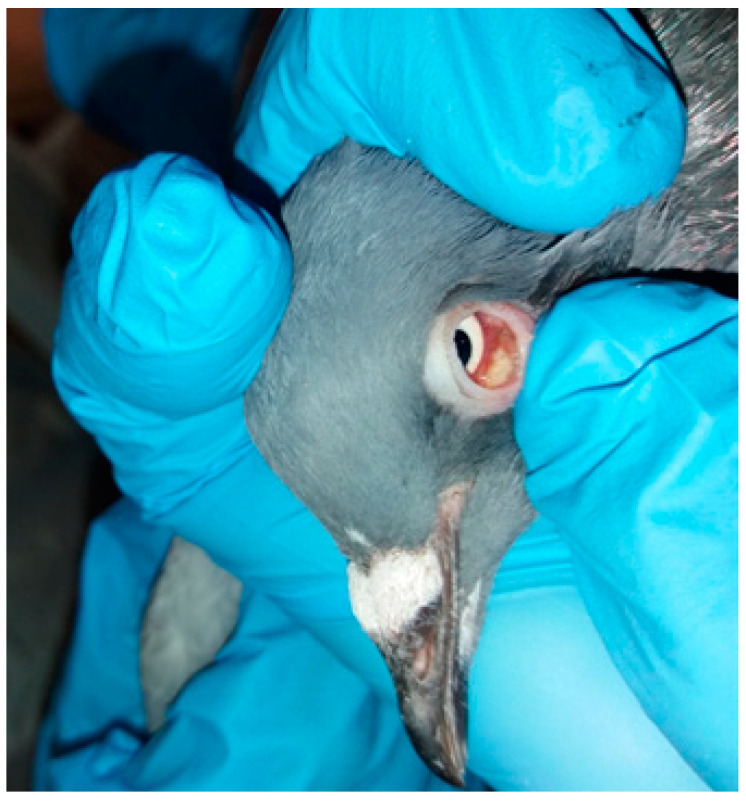
Nodular lesions in the conjuctiva of the inferior left eyelid in a racing pigeon.

**Figure 2:**
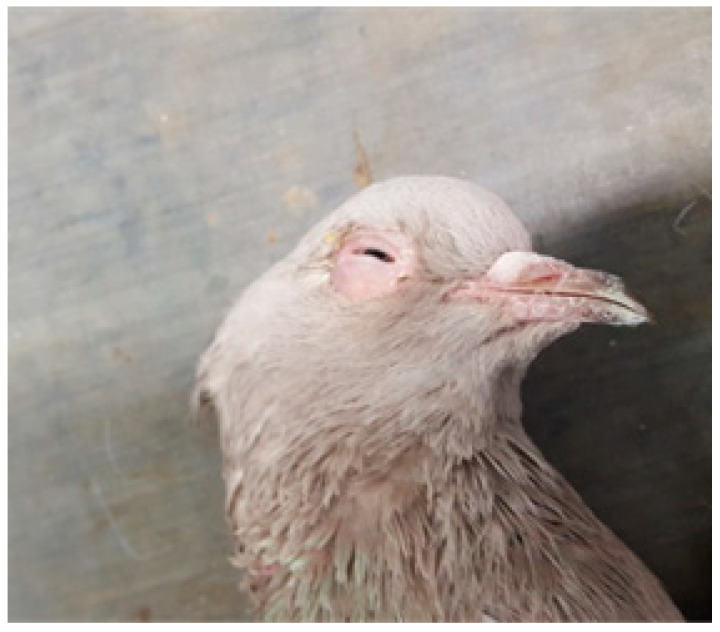
Ocular secretions leading to stuck and loss of feathers around the right eye in a racing pigeon.

Three and four racing pigeons from two different lofts, respectively, were submitted to the Unit of Avian Medicine, Clinic of Farm Animals, Faculty of Veterinary Medicine, Aristotle University of Thessaloniki in Greece. The first loft (A) consisted of 120 racing pigeons with ages from 1 day until 5 years old. Three racing pigeons 2 years old were submitted alive for clinical and post mortem examination. Based on the records, the clinical signs started in the loft one year before, while the clinical signs in the pigeons were observed 1 month before their submission in our clinic. The second loft (B) consisted of 400 racing pigeons, with ages from 1 week until 10 years old. Four racing pigeons, with ages of 4–5 years old were submitted alive for clinical and post mortem examination in our clinic. The clinical signs had started 5 to 6 years ago in the loft. The signs of the submitted pigeons from both lofts were variable and not pathognomonic of the disease. The submitted pigeons exhibited signs of diarrhea, polyuria, dehydration, lameness and flying inability. In addition, they had wet eyes with yellow mucus, swelling of the inferior eyelids and loss of feathers around the eye (Figure 1 and Figure 2). The nutritional status was poor, evident as atrophy of pectoral muscles with a prominent keel, although the appetite was normal. According to the history, their diet was based on corn and bread, while twice a week various vegetables were also provided. Fresh water was provided every day.

During visits to the affected flocks the health status and the biosecurity measures were evaluated. In particular, 3–5% birds of the flocks exhibited clinical signs similar to the submitted birds while the rest of the flocks seemed healthy. As it concerns the biosecurity of the lofts, wild birds and backyard chickens were in contact with the racing pigeons at both sites. The size of the lofts was small for the population of the pigeons with a lot of dust and droppings, which contaminated the feeders and the drinkers.

## 3. Post Mortem Examination

**Figure 3:**
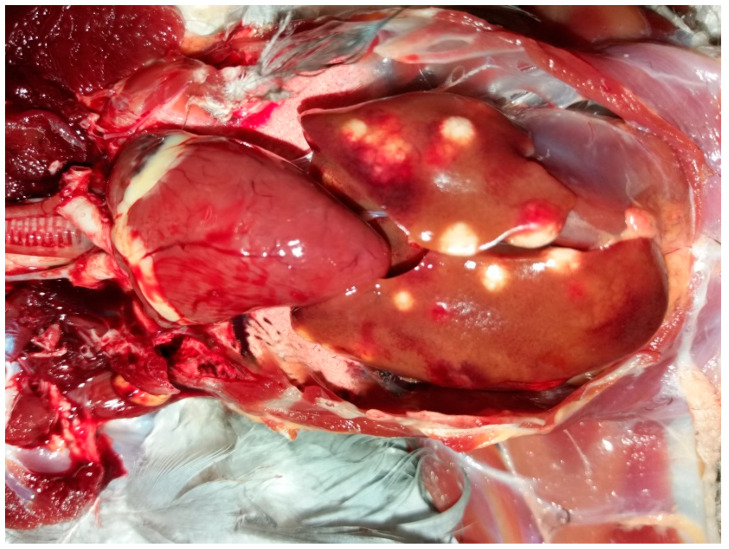
Granulomatous lesions in the liver of a racing pigeon.

The pigeons were humanely euthanized and then necropsied. They were in poor nutritional status accompanied with cachexia. The post mortem examination revealed enlarged pale spleen and liver with lots of characteristic white-yellow nodules of difference sizes (Figure 3). Similar nodules were observed inside of the inferior and superior eyelids as well as in the bone marrow of the right femur. The pigeons of the loft B had similar but more severe lesions, as described in the loft A. Tissue samples from affected organs of the pigeons from both lofts were taken for microbiological and histopathological examination as well as for molecular identification.

## 4. Microbiological Examination

Samples from the inferior and superior eyelids, liver, spleen and bone marrow of the right femur were cultured in blood agar and MacConkey agar in aerobic and anaerobic conditions, as well as in Sabouraud dextrose agar but no pathogenic bacteria or fungi were isolated. However, the presence of a large number of acid-fast bacilli was revealed in the Ziehl-Neelsen stained smears.

## 5. Histopathological Examination

**Figure 4:**
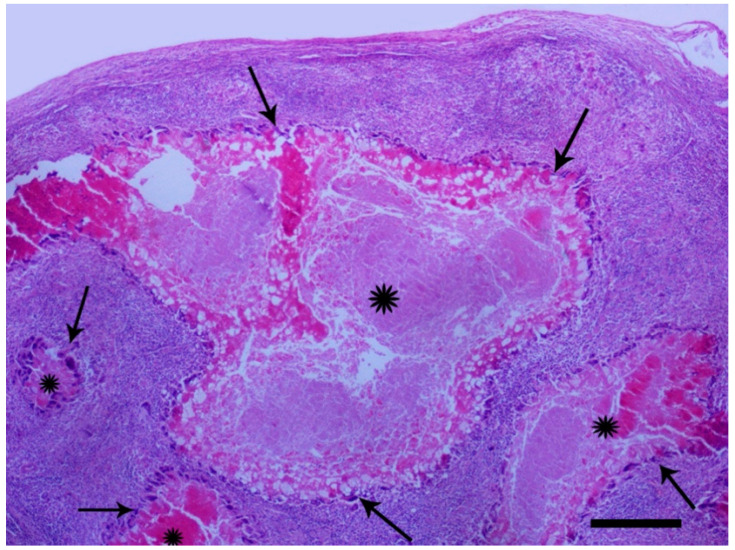
Racing pigeon, spleen, four granulomas ranging in size, with variable degree of central necrosis (asterisks) are seen. The necrotic areas are surrounded by a basophilic layer of epithelioid macrophages and giant cells (arrows). H&E, bar = 250 μm.

**Figure 5:**
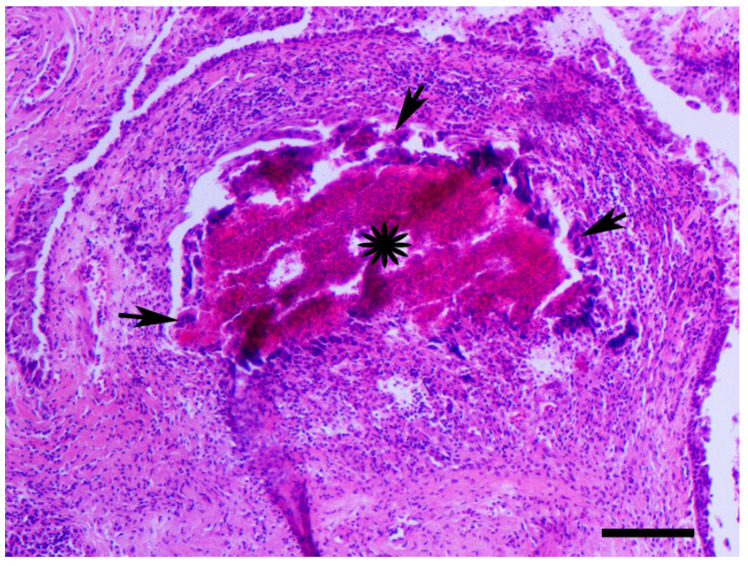
Racing pigeon, eyelid, the underlying lamina propria of the mucosa has a large granulomatous area with central necrosis composed of amorphous eosinophilic material intermingled with cell remnants (asterisk). This necrosis is surrounded by a rim of Langhans type giant cells (arrows) and epithelioid macrophages. Peripherally to the above cells numerous lymphocytes are obvious. H&E, bar = 100 μm.

Tissue samples from the spleen, liver and eyelids were fixed in 4% buffered formaldehyde for 48–72 h. Coronal sections from the samples were embedded in paraffin by a routine procedure. Dewaxed 3–5 μm thick sections were stained with hematoxylin and eosin (H&E). The presence of multiple granulomas consisted of central necrosis of varying degrees surrounded by a thick layer of epithelioid macrophages and multinucleated giant cells of Langhans type was observed. At the periphery of that zone dominated lymphocytes and in certain nodules, an outer layer of connective tissue was observed (Figure 4 and Figure 5).

**Figure 6:**
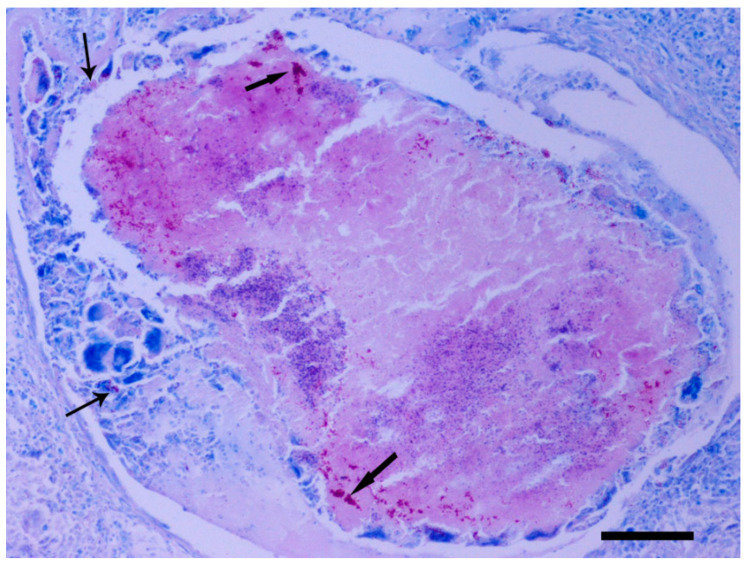
Racing pigeon, eyelid, intensely eosinophilic rods (acid-fact bacilli) single or forming clumps are primarily seen within necrosis (thick arrows) and in the cytoplasm of Langhans type giant cells (thin arrows). Z-N, bar = 100 μm.

Ziehl-Neelsen histochemical staining was also performed on serial sections of the above-mentioned tissue samples and acid-fast bacilli were found within the cytoplasm of macrophages and giant cells. Similar bacilli single or in clusters, was observed lying free amongst inflammatory cells and in the necrotic area as well (Figure 6).

## 6. Molecular Identification

DNA was isolated from nodular lesions from liver, spleen, bone marrow and inferior eyelids, using a phenol-chloroform-isoamyl alcohol extraction protocol as previously described [22]. Polymerase chain reaction (PCR) assays were carried out for the identification of the specific insertion sequences of IS1245 and IS901 according to a previously described method [17]. The presence of IS1245 and IS901 was identified in the submitted samples. Since IS901 is present in both MAA and MAS [17], DNA extracts were further tested for the specific identification of MAA strains according to the real-time PCR method developed by Ronai et al., [21] and revealed the presence of MAA. The targeted putative membrane protein gene amplicon was sequenced for verification reasons revealing the CG polymorphism characterizing MAA strains [21].

## 7. Discussion

Avian mycobacteriosis is an important disease with increasing economic consequences. It is common in backyard poultry, wild, zoo and aviary birds but rare in commercial poultry. Pigeons were considered to be highly resistant to infection but in the last decade pigeon mycobacteriosis has become an emerging threat for pigeon flocks around the world [1,23].

In our case study, avian mycobacteriosis occurred in pigeons over 2 years of age, with chronic progression and muscular atrophy. The clinical signs were prolonged for months and multiple nodules were observed in the liver, spleen, eyelids and bone marrow. The disease is less prevalent in young pigeons due to the long incubation period needed for the outbreak of the disease [1].

Nodules are most frequently present in the liver, spleen, intestines and bone marrow. Some organs, such as heart, ovary, testes and skin, are infrequently affected and cannot be considered organs of predilection [7]. However, in our case study we observed nodular lesions in the inferior and superior eyelids. The location of the lesions is affected by the route of infection. In particular, the location of the lesions in the liver, spleen and intestine may reflect oral acquisition and suggest a mycobacterial intake in contaminated food and water, whilst lesions in the lungs and other areas of the respiratory tract suggest inhalation as being the exposure route [24]. The eyelids lesions may result from entry of the organism through abrasions in the skin around the eye [8].

Swollen eyelids with conjunctivitis must be differentiated from other diseases or conditions, which could cause similar ocular lesions. These include bacterial infections by *Chlamydophila psittaci*, *Mycoplasma* spp., *Pasteurella* spp., *Listeria* spp., *Staphylococcus* spp., *Streptococcus* spp., *Escherichia coli*, *Pseudomonas* spp., *Klebsiella* spp., *Citrobacter* spp. and *Haemophilus* spp., viral infections by avian poxvirus or herpesvirus, parasitic infections by *Oxyspirura* spp., *Philophthalmus* spp. and *Thelazia* spp. and mycotic infection by *Candida* spp. In addition, neoplasia, trauma and hypovitaminosis A can also cause lesions in the conjunctiva. The history and the clinical examination of the affected birds as well as the results of the histopathological and bacteriological examinations were essential to differentiate avian mycobacteriosis from other reported diseases [25,26,27,28]. The smears stained with Ziehl-Neelsen from the lesions of the eyelids confirmed the disease.

It is difficult to determine the origin of infection in either affected pigeon lofts. Wild birds usually serve as major reservoirs which are responsible for the shedding of the *M. avium* subsp. *avium* to the environment and facilitating its spread for years [1]. Additionally the contact with backyard chickens is a very probable cause of infection [11]. Based on the history of our case study, free-living birds, foreign visitor pigeons and backyard chickens were in contact with the pigeon lofts and they could be the source of infection. Moreover, the poor hygienic standards, the overpopulation, the multiage flock as well as the inadequate nutrition could have contributed to the establishment of the disease.

Mycobacteriosis is a List B disease of the World Organization for Animal Health. However, *Mycobacterium avium* and *M. genavense* can cause disseminated infections in AIDS patients and otherwise immunocompromised patients, that is refractory to treatment [1]. They can also cause lymphadenitis in mammals and similar lesions such as paratuberculosis (Johne’s disease) in ruminants [5,12]. The diagnosis of avian mycobacteriosis and the identification of the causative agent are very important for the control of the disease in birds and to protect other animals and humans, because pigeons are usually kept in urban areas for homing and racing purposes. The molecular analysis revealed a typical MAA strain of genotype IS901+ and IS1245+. This genotype can cause serious disease in mammals such as cattle, horse sheep, pig and humans [17]. However birds seem to be more sensitive to infection [29,30].

Treatment of avian mycobacteriosis in birds is not recommended, because of the high risk of resistance to the antituberculosis drugs, which are also used in human medicine. Moreover, it is considered of high cost and prolonged time, which usually takes up to 12–18 months [31,32]. The eradication of *M. avium* infection is difficult due to the chronic carrier state and intermittent shedding of organisms by the infected birds. *Mycobacterium* spp. can survive for over 4 years in the environment and is resistant in extreme weather conditions as well as to many disinfectants [1]. Dhama et al., [12] recommended sacrificing the affected flocks, abandoning the equipment and housing materials, removal of litter and contaminated soil, elimination of older flocks, followed by strict biosecurity procedures. In our case, we suggested the owners to remove all the contaminated equipment and materials, to eliminate the contaminated flocks and to establish the new flocks on a different location with strict biosecurity conditions, preventing the contact with the old contaminated environment.

Inactivated and/or live experimental vaccines against avian mycobacteriosis have been used in birds, which decreased the severity of the lesions in the liver but they did not prevent the infection [33]. Encouraging results have been shown in chickens after oral vaccination with live *M. intracellulare* serovar 6 (*M. avium* serovar 6), as well as the combination of intramuscular vaccination with inactivated plus live *M. intracellulare* serovar 7 and serovar Darden (*M. avium* serovars 7 and 19) [1,32].

In our cases the DNA sequencing presented the genotype IS901+ and IS1245+ simultaneously in the tissue samples. The extracted DNA was also tested with the real-time PCR method for the identification of MAA strains. This case study is the first report of molecularly identified MAA in pigeons in Greece. Furthermore, the granulomatous conjunctivitis has not been described in cases of pigeon mycobacteriosis in Greece either and it should be considered in the differential diagnosis. The identification of the strain will enrich the epidemiological data and will contribute to the development of a homologous vaccine, in order to control avian mycobacteriosis in pigeons.

## Data Availability

None of the data were deposited in an official repository.
